# Correction: Single-molecule nanopore sensing of actin dynamics and drug binding

**DOI:** 10.1039/d0sc90132f

**Published:** 2020-07-23

**Authors:** Xiaoyi Wang, Mark D. Wilkinson, Xiaoyan Lin, Ren Ren, Keith R. Willison, Aleksandar P. Ivanov, Jake Baum, Joshua B. Edel

**Affiliations:** Department of Chemistry, Imperial College London, Molecular Sciences Research Hub White City Campus 80 Wood Lane W12 0BZ UK joshua.edel@imperial.ac.uk; Department of Life Sciences, Imperial College London Sir Alexander Fleming Building, Exhibition Road, South Kensington London SW7 2AZ UK jake.baum@imperial.ac.uk

## Abstract

Correction for ‘Single-molecule nanopore sensing of actin dynamics and drug binding’ by Xiaoyi Wang *et al.*, *Chem. Sci.*, 2020, **11**, 970–979, DOI: 10.1039/C9SC05710B.

In the original article, the authors regret that an inappropriate estimate of the effective length was used. The method used by the authors in the original manuscript (Fig. S9 and Method S4) is only suitable for 1D solid-state nanopores rather than nanopipettes. This has now been corrected from 110 nm to 45 nm based on a previous paper by the authors.^1^ Due to this correction, some calculated values, some text in the main article, some figures, and some methods have changed. There was also an error in the calculation of excluded volume that has been corrected in the new values. The changes are as outlined below. These revisions do not alter the scientific conclusions of the manuscript.

(1) On page 972, the text should read: “The *H*_eff_ of the nanopipette was determined to be 45 nm based on our previous measurements”.

(2) On page 973, the text should read: “The distribution is not a standard Gaussian distribution as part of the low translocation signals were cut off by the low-pass filter, which we also see for the normalised current blockade (Δ*I*/*I*_o_) shown in Fig. S9”.

(3) On page 973, the text should read: “The plot of mean excluded volume *vs.* urea concentrations exhibits a sigmoidal shape and increases from 50.7 ± 4.2 nm^3^ to 73.6 ± 6.2 nm^3^ and corresponds to a two-state transition from folded to unfolded actin (Fig. 2b)”.

**Fig. 2:**
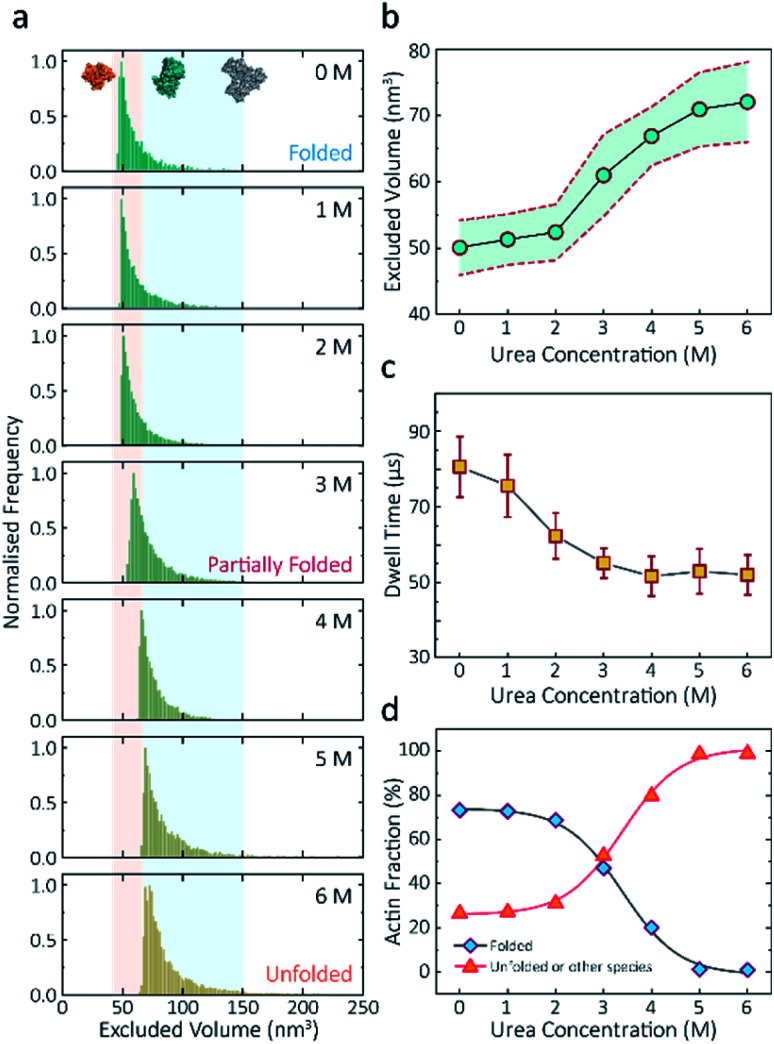
Discrimination of actin unfolding in a urea gradient using nanopipettes. (a) Normalised statistic of excluded volumes (calculated from *Λ* ≈ Δ*I*_b_*H*_eff_^2^/(*σψ*)) from native actin to denatured actin in increasing urea concentration. The orange boundary represents the fully folded actin state, and the blue boundary determines the unfolded actin or other transient aggregates at low urea concentrations. The population shifts from a mostly native, folded state to a higher excluded volume, consistent with an unfolded state with larger hydrodynamic radius, as the urea concentration increases. Protein models for each state based on the crystal structures of G-actin (PDB: 1NWK) are shown in the inset. (b) The mean excluded volume plot exhibits a sigmoid increase when the urea concentration increases, indicative of actin unfolding with an increase of hydrodynamic radius. (c) Mean dwell time decreases as the urea concentration increases. (d) Proportion of actin in different states (monomeric folded, unfolded or transient aggregated) plotted against urea concentrations shows a sigmoid curve, suggesting a two-state transition between folded (including a small fraction of aggregates) and unfolded actin.

(4) On page 973, the text should read: “Based on the assumption that actin is fully unfolded at 6 M urea, a threshold of 67 nm^3^ for the excluded volume at 6 M was used to define the transition between folded (<67 nm^3^) and unfolded (>67 nm^3^) actin. This value is consistent with the reported value at 0 M urea with no applied voltage as we see a slight decrease in excluded volume upon addition of voltage (Fig. S11)”.

(5) On page 973, the text should read: “By this linear extrapolation, we can determine the free energy of actin unfolding to be 7.74 kJ mol^−1^ at 25 °C without any denaturant (Method S4 and Fig. S10)”.

(6) On page 974, the text should read: “By plotting effective velocities (*ν*_eff_ = *H*_eff_/*t*_d_) *vs.* applied voltages, we can extract the slope (**∂***ν*_eff_/**∂*Ψ***) and therefore give the zeta potential of actin using an available model (−6.0 mV for folded actin and −12.1 mV for unfolded actin, Method S5)”.^35^

(7) On page 975, the text should read: “We assume the protein forms a hard-sphere during nanopore translocation (Methods S4 (ref. 38)). Distributions of Δ*I*/*I*_o_ for folded and unfolded actin at different voltages are shown in Fig. S11, where we can observe a decrease in Δ*I*/*I*_o_ as the voltage increases. The mean calculated *R*_H_ and excluded volume as a function of applied voltages are shown in Fig. S12”.

(8) On page 975, the text should read: “We calculated *p* values for folded actin ranging from 0.999 ± 0.019 at 150 mV to 1.178 ± 0.071 at 350 mV (Fig. 3e), suggesting the protein is approximately defined as an oblate ellipsoid, in agreement with the stretching of proteins with a dipole moment under an electric field.^23^*p* for unfolded actin also exhibits a similar trend increasing from 1.825 ± 0.050 to 2.072 ± 0.121”.

**Fig. 3:**
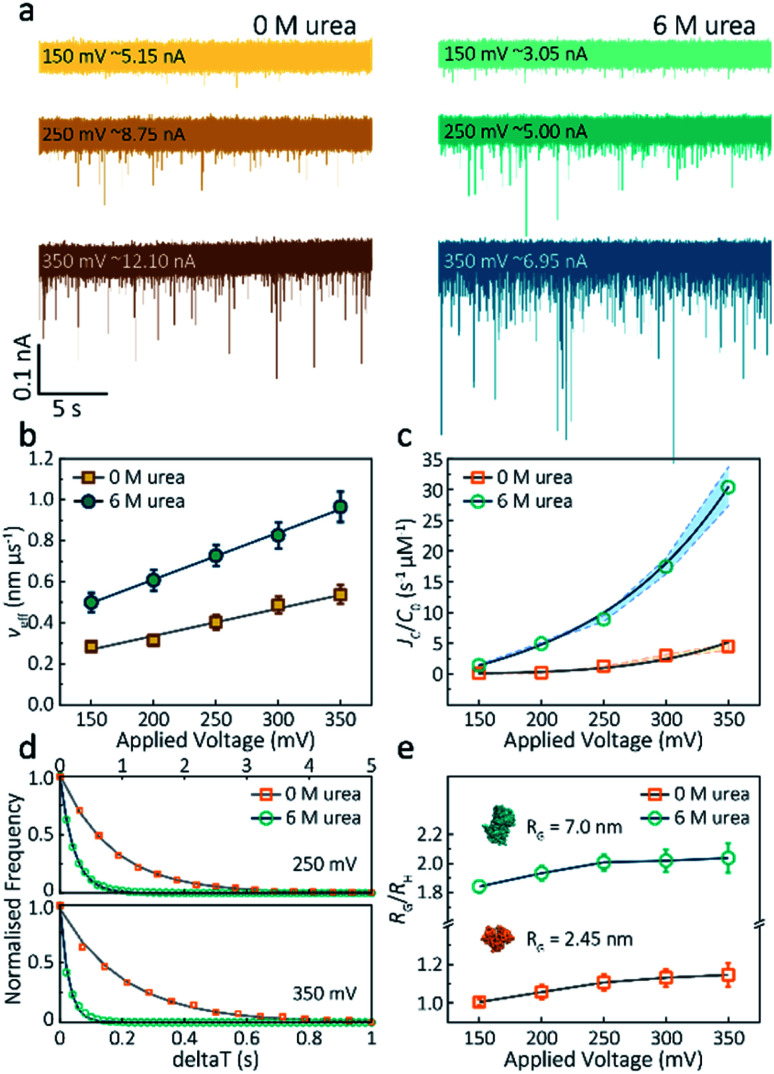
Voltage dependence upon actin translocation through nanopipettes. (a) Voltage-dependent ionic current traces show the translocation spikes of 800 nM folded actin in 0 M urea and unfolded actin in 6 M urea. Open currents (*I*_o_) for each voltage were marked upon each baseline. (b) Effective velocity (*H*_eff_/*t*_d_) for both folded and unfolded actin shows a linearly voltage-dependent increase when increasing the applied voltage. (c) Normalised capture rates (*J*_C_/*C*_0_) show an exponential function of applied voltage for both folded and unfolded actin. This nonlinear increase suggests a two-stage regime in which the entropic barriers restrict successful translocations at low voltages and electrophoretic forces dominate capture behaviours at higher voltages. (d) Normalised distributions of elapsed time between successive captured events (*δt*) for actin transport in 0 M and 6 M urea buffers at 250 mV and 350 mV. Solid lines represent a single-exponential decay fit, from which the protein flux (*J*_C_) is extracted (for c). (e) Plots of the ratio of the radius of gyration to hydrodynamic radius (*R*_G_/*R*_H_) illustrate that the actin shape is an oblate ellipsoid in 0 M urea buffer, but a prolate ellipsoid in 6 M urea buffer during nanopore translocation. With voltage increase, the value of *R*_G_/*R*_H_ increases both for folded and unfolded actin. This is unchanged at high voltages, indicating the protein was stretched under a high-strength localised electric field across the pipette tip.

(9) On page 975, the text should read: “The distribution of peak current in Fig. S13 shows time-dependent multiple populations, indicative of a concomitant increase in both the proportion and degree of ATP-actin polymerisation”.

(10) On page 975, the text should read: “By measuring the IV curves before and after nanopore experiments (Fig. S14), we can be certain these changes do not originate from the interaction between the analytes and the nanopore and thus are directly related to filament formation”.

(11) On page 977, the text should read: “Upon addition of excess Latrunculin B, the peak current of translocation events became more uniform at different voltages (59.1 ± 5.4 pA compared with native monomers of 64.8 ± 6.7 pA at 250 mV, Fig. S13)”.

(12) On page 977, the text should read: “Incubation with Swinholide A almost doubles the mean peak current from 59.1 ± 5.4 pA to 113.7 ± 15.8 pA (at 250 mV, Fig. S16) and increases the capture frequency from 1.58 ± 0.08 s^-1^ to 5.07 ± 0.16 s^−1^, while no observable difference can be seen between the capture frequencies of monomeric actin with or without Latrunculin B”.

(13) On page 977, the text should read: “The electrokinetic properties of actin monomers and dimers, including dwell time and capture rate, are shown in Fig. S16 and 5d”.

(14) Fig. 2a and b, and Fig. 3b and e have been updated to reflect these changes. The *x* axis titles for Fig. 2a and d have also been included as they were missing in the original manuscript. The corrected figures are shown:

(15) In the ESI file, Method S4 and Fig. S9 have been deleted, and Fig. S12 and S13 have been corrected. The order of the methods and figures has also been revised. Please see the updated ESI for these changes.

These revisions do not alter the scientific conclusions of the manuscript.

The Royal Society of Chemistry apologises for these errors and any consequent inconvenience to authors and readers.
